# Efficacy of X-Ray Phytosanitary Irradiation on the Infectivity and Reproduction of *Angiostrongylus cantonensis* in Experimentally Infected Rats

**DOI:** 10.4269/ajtmh.23-0570

**Published:** 2024-02-20

**Authors:** Susan I. Jarvi, Lisa M. Kaluna, Steven C. Hess, Lindsey Hamilton, Carmen Antaky, Robert T. Sugihara, Israel L. Leinbach, Yaeko Tagami, Argon Steel, Kathleen Howe, Steven Jacquier, Forrest Cookman, Jocelyn Diaz, John Jacob, Peter Follett

**Affiliations:** ^1^ Department of Pharmaceutical Sciences, Daniel K. Inouye College of Pharmacy, University of Hawaii, Hilo, Hawaii;; ^2^ U.S. Department of Agriculture, Animal Plant Health Inspection Service, National Wildlife Research Center, Hawaii Field Station, Hilo, Hawaii;; ^3^ U.S. Department of Agriculture, Agricultural Research Service, Daniel K. Inouye Pacific Basin Agricultural Research Center, Hilo, Hawaii

## Abstract

*Angiostrongylus cantonensis* is a globally distributed nematode and the leading cause of eosinophilic meningitis in humans. As a global hotspot for this disease, Hawaii’s agricultural exports may be contributing to the spread of *A. cantonensis*. Phytosanitary irradiation doses of 150 or 400 Gy provide quarantine security against multiple insect pests. We evaluated the in vitro and in vivo effects of phytosanitary irradiation on infectious, third-stage, *A. cantonensis* larvae. In vitro experiments directly exposed larvae to irradiation doses ranging from 200 to 1,000 Gy. Results showed low mortality and no dose response across all treatments 27 days post-irradiation. In vivo studies isolated larvae from wild-caught *Parmarion martensi* after exposure to x-ray irradiation at doses of 0, 150, and 400 Gy and infected them into laboratory rats. Fourteen rats were assigned to each treatment and infected with 50 larvae from their assigned irradiation dose. Results at 3 and 6 weeks post-infection demonstrated a significant negative dose response in regard to the number of larvae that migrated to the brain and adults found in the pulmonary artery. No irradiated larvae that grew into adults were able to produce eggs. These findings indicate that x-ray irradiation does not result in the direct mortality of *A. cantonensis* larvae; however, it does affect the infectivity and reproduction of *A. cantonensis* within its definitive host, the rat. Phytosanitary irradiation at doses ≥150 Gy appears to be an effective means of preventing the establishment of viable populations of *A. cantonensis*, thus reducing the potential for global spread due to agricultural exports from Hawaii.

## INTRODUCTION

Neuroangiostrongyliasis (rat lungworm disease) is caused by the parasitic nematode *Angiostrongylus cantonensis*. The definitive host is the rat (primarily *Rattus* spp.), and completion of the life cycle of *A. cantonensis* requires an intermediate gastropod host (slugs and snails). First documented in Canton, China, in 1935, by *1944 A. cantonensis* was known to cause human disease.^1^ Intentional or accidental ingestion of *A. cantonensis* contaminated food or water is thought to be the primary means of human infection.^2^^–^^5^ Additionally, paratenic hosts such as crustaceans, frogs, lizards, and planarians can also cause human infection.^6^ Outbreaks of eosinophilic meningitis attributed to *A. cantonensis* were reported in the Pacific Islands by the early 1960s.^7^ This parasite now has a global distribution, including the continental United States.^8^^–^^13^

Phytosanitary irradiation doses of up to 1,000 Gy (the unit of absorbed radiation energy) have been approved by the U.S. Food and Drug Administration to control quarantine pests and disinfest produce to protect U.S. agriculture, the environment, and importing economies from invasive plant and animal pests and diseases.^14^ The U.S. Department of Agriculture (USDA) approved 150- and 400-Gy doses for tephritid fruit flies and other insects, the International Plant Protection Convention has only adopted the 150-Gy dose for quarantine security.^15^ Irradiation easily penetrates food products, and regulatory protocols require that the minimum absorbed dose, either 150 or 400 Gy, is achieved throughout the product during commercial treatment.^15^ X-ray irradiation is used to control quarantine insect pests on some fresh fruits and vegetables exported from Hawaii to the U.S. mainland. With snails acting as intermediate hosts to *A. cantonensis*, one way to impede the establishment of viable parasite populations is to control species known to be efficient carriers of the nematode. *Parmarion martensi*, colloquially known as the semi-slug, is an abundant and efficient carrier of *A. cantonensis*, which has been found on Hawaii produce (e.g., sweet potatoes, taro leaves) exported to the U.S. mainland and was prioritized as a quarantine pest of concern in 2009 by the USDA.^16^ We have previously completed a study to determine whether irradiation can inhibit *P. martensi* reproduction and thereby reduce the risk of the establishment of *P. martensi* on the U.S. mainland and reduce the potential for transmission of *A. cantonensis*.^17^^,^^18^ Follett et al.^18^ found irradiation at 150 and 400 Gy caused reduced growth, a higher rate of mortality, and prevented reproduction in *P. martensi*. Their results were consistent with other snail irradiation studies, concluding that the 150-Gy generic dose may be effective against many snail species. However, the inability of a snail to establish viable populations may only retard the *A. cantonensis* lifecycle if a rat or other viable intermediate host consumes the snail,^19^ unless irradiation also affects the parasite within. The effects of irradiation on the life cycle of *A. cantonensis* within its definitive host have not been thoroughly examined with these generic doses.

Four studies have been conducted to evaluate the infection of irradiated *A. cantonensis* in rats. Lee^20^ was the first published report evaluating the antigenicity and immunogenicity of larvae irradiated at 20 and 40 Kr (200 and 400 Gy). Although multiple experiments were performed, they reported that infection was present in one of six rats infected with 400 larvae treated with 40 Kr (400 Gy) and two of two rats infected with 200 larvae treated with 20 Kr (200 Gy). They also reported that a dose of 20 Kr (200 Gy) resulted in the inability of young adult worms to reproduce. Ishii et al.^21^ irradiated first-stage (L1) *A. cantonensisi* larvae at doses ranging from 500 to 10,000 R (5–100 Gy) allowed them to develop to L3 in intermediate hosts, and then infected rats with the L3 larvae. They found a negative dose response in terms of overall recovery of adult worms, with some infection observed across all treatments. Their maximum dose of 100 Gy resulted in low infection levels and prevented reproduction with no eggs or L1 observed. After irradiating *A. cantonensis* infected snails at doses ranging from 250 to 1,000 Gy, Pai et al.^22^ infected rats and mice (a nonpermissive host) with both irradiated and nonirradiated L3 larvae. A dose of 250 Gy was sufficient to prevent the production of eggs in rats, and a dose of 500 Gy resulted in no worms or pathologic findings found in the heart–lung complex of rats, or brain tissue of mice. Ooi et al.^23^ only reported on infection levels in mice and rat brains or spinal cord after infection with x-ray– and gamma-ray–irradiated L3 larvae of *A. cantonensis* and a related species, *A. costaricensis*, at doses ranging from 500 to 4,000 Gy; no findings related to reproduction were reported. Necropsies occurring approximately 1-week postinfection (PI) found a dose response with a minimal dose of 2,000 Gy needed to inhibit infectivity. Overall, these studies show conflicting results regarding the irradiation dose required to prevent infection of *A. cantonensis* in rats completely but were consistent in that doses ≥100 Gy prevented reproduction with a lack of eggs or L1 larvae.

Although research has documented some species of nematodes to be quite tolerant of irradiation,^24^ the previous studies on *A. cantonensis* (summarized earlier) suggest that the USDA quarantine security dose of 150 or 400 Gy would be effective for quarantine security of this species, which is often accomplished by preventing the reproduction of a pest. The goal of this study is to evaluate the efficacy of x-ray irradiation in limiting *A. cantonensis* infectivity and reproduction in its definitive rat host at doses recommended for agricultural exports (150 and 400 Gy) from Hawaii. Necropsies at 3 and 6 weeks PI, as well as polymerase chain reaction (PCR) analysis on brain and feces tissues allow for a more in-depth analysis of infection than previous studies.

## MATERIALS AND METHODS

### In vitro irradiation of *A. cantonensis* larvae.

*Angiostrongylus cantonensis* larvae were isolated using techniques by Howe et al.^5^ from wild *P. martensi* semi-slugs that were collected from east Hawaii Island. Semi-slugs were drowned in 50-mL Falcon tubes (Falcon, Corning, NY) of municipal (tap) water in an air-conditioned laboratory kept at 22°C. After 3 days, nematodes with morphology and swimming behavior consistent with the *A. cantonensis* third-stage larvae (L3)^25^ were identified using stereoscopes (Leica Microsystems S9D, Deerfield, IL and Wild Heerbrugg M4A APO, HeerBrugg, Switzerland). Larvae were individually isolated from the water by pipette and placed in a glass Petri dish of fresh tap water. In total, 9,000 larvae were pooled, mixed, and “washed” twice with tap water to remove bacteria and other debris from the water as well as for better oxygenation. Washes were simply the transfer of larvae into fresh water by pipette, using as little water as possible from the previous dish to reduce debris. Early testing also showed that removal of debris was helpful in visualizing larvae during subsequent analysis with the propidium iodide assay.^26^

Larvae were divided into five technical replicates of 200 larvae for each treatment as independent measures of the x-ray irradiation treatment. Larvae destined to be x-ray irradiated were transferred into 15-mL conical Falcon tubes with 7 mL of tap water for each technical replicate. Three optichromic dosimeters (FWT-70-83M, Far West Technology, Goleta, CA) were used to confirm each irradiation dose, submerged in 7 mL of tap water in a 15-mL Falcon tube. An electron linear accelerator (5 MeV, model TB-5/15; Titan Corp., San Diego, CA) located at the Hawaii Pride commercial x-ray irradiation facility in Keaau, Hawaii, was used for irradiation. This facility was designed to apply low-dose irradiation for phytosanitation of fresh agricultural produce. The target irradiation doses were 0 (unirradiated control), 200, 400, 600, 800, and 1,000 Gy. To control dose uniformity (the ratio of the maximum/minimum dose), the test tube rack holding a single row of upright Falcon tubes with five larvae technical replicates and three dosimeters were placed perpendicular to the x-ray beam and elevated by placement on a fiberboard box and positioned in the center of the carrier for treatment at each dose. The dosimeters were placed at each end and in the middle of the rack, between the tubes of larvae. After irradiation treatment, dosimeters were read with an FWT-200 reader (Far West Technology) at 620-nm absorbance to measure dose accuracy and variation. Mean measured doses (plus range) were 204.1 (189–225), 420.9 (417–424), 603.3 (598–607), 812.0 (799–821), and 978.5 (969–986) Gy.

Parasite mortality was monitored for 23 days postirradiation using a propidium iodide assay.^26^ After irradiation, larvae were transferred into a 384-well glass-bottom plate (PerkinElmer, 6007460, Waltham, MA) in a total volume of 120 µL of tap water and a final concentration of 1× penicillin–streptomycin (Omega Scientific, PS-20, Tarzana, CA) and 1.25% propidium iodide stain (Biotium, 40017, Freemont, CA). Unirradiated larvae were used for live controls, dead controls, and genetic analysis. Methanol-frozen dead control larvae were used to confirm staining and visualization procedures.^26^ The plate was imaged every 2 to 3 days on an Operetta High Content Imaging System (Perkin Elmer, HH12000002) to check for propidium iodide staining of the larvae. Penicillin–streptomycin was added every 2 to 3 days to minimize bacteria growth, and the wells with notable water loss from evaporation were topped off. The plate of larvae was stored between imaging in a dark cabinet at an average temperature of 19.8°C with 74.7% relative humidity.

A few larvae in each treatment of the plate were observed moving under a stereoscope at 27 days postirradiation. All larvae were subsequently removed from the 384-well plate and transferred to a gridded Petri dish with 5 mL of fresh tap water, one dish per technical replicate. Each Petri dish was incubated under a stereoscope with a substage light for 5 minutes, and then the number of moving larvae was counted. To stimulate movement, 5 mL of a1% pepsin and 1.4% HCl solution was added to each Petri dish and swirled for a final concentration of 0.5% pepsin–0.7% HCL solution.^5^ The number of moving larvae was observed after a 5-minute incubation period in the pepsin-HCl solution.

### In vivo irradiation of *A. cantonensis* and isolation from *P. martensi* (semi-slug).

Potentially infected wild semi-slugs (*N* = 148) were collected with permission from the landowners of nine locations on Hawaii Island (4–31 individuals per site) and reared for 1–3 days before irradiation at the University of Hawaii at Hilo Daniel K. Inouye College of Pharmacy according to Hamilton et al.^17^ Semi-slugs from each collection site were divided as evenly as possible into rearing containers for the three irradiation dosage treatment groups of 0 Gy (*n* = 48), 150 Gy (*n* = 49), and 400 Gy (*n* = 51).

For treatment with irradiation, 2 to 3 semi-slugs were placed in Corning Falcon 50-mL conical centrifuge tubes with ventilated caps and transported to a commercial irradiation facility (RLH Hawaii Pride, Keaau, Hawaii) for treatment with x-rays using an electron linear accelerator (5 MeV, model TB-5/15; Titan Corp., San Diego, CA). To optimize dose uniformity (DUR, the ratio of the maximum/minimum dose), Falcon tubes with semi-slugs were placed in the center row of a 3 × 8 test tube rack which was elevated by placement on a plastic box (20 cm height) placed perpendicular to the x-ray beam and positioned in the center of the carrier. Dose mapping was conducted at 150 and 400 Gy with empty Falcon tubes to determine dose variation during treatment. For each dose, four optichromic dosimeters (FWT-70-83M, Far West Technology) were attached to the outer surface at four compass points around each of the three tubes positioned at the ends and in the middle of the test tube rack. Semi-slugs usually crawled to the tops of the tubes, so dosimeters were attached around the upper portion of the tubes. After determining the belt speed to deliver the target doses of 150 and 400 Gy from dose mapping, tubes with the semi-slugs were inserted into the test tube racks for irradiation treatment. During irradiation treatment of semi-slugs, three dosimeters were interspersed along the line of test tubes to confirm dose delivery at the predicted level, for example, within the range of doses determined from the extensive dose mapping. Optichromic dosimeters were read with an FWT-200 reader (Far West Technology) at 620-nm absorbance. The belt speed for the 150- and 400-Gy treatments was 0.366 m/minute and 0.143 m/minute, respectively, and the test tubes containing semi-slugs were treated from one side and then the other in a two-pass treatment. The mean measured doses (plus range) in the 150- and 400-Gy treatments were 155.9 (range 142–164_._) Gy and 397.0 (range 372–412) Gy for a DUR of 1.11 to 1.15. Semi-slugs were returned to their rearing containers after irradiation for 24 hours until they were drowned in tap water in 50-mL Falcon tubes, individually, for larvae isolation after 3 days.^5^

On the fourth day post-irradiation, larvae were isolated from the water of the drowned semi-slugs at a designated station to avoid cross-contamination between treatments. Larvae were isolated in two ways; both individually using a pipette into glass Petri dishes with tap water and using a Baermann’s funnel filled with tap water. Overall, larvae were isolated from 16 semi-slugs of the 0-Gy treatment, and 15 semi-slugs of both the 150- and 400-Gy treatments; no larvae were found in the water of the other drowned semi-slugs. Larvae were pooled by treatment and “washed” to minimize debris and bacteria in the surrounding water column by individually isolating and transferring larvae to a new Petri dish of tap water by pipette. Only nematodes that resembled the morphology and swimming behavior of the third-larval stage (L3) of *A. cantonensis* were isolated.^25^ An antibiotic-antimycotic solution (Sigma A5955-100ML, Livonia, CA) was added to the pool of larvae to avoid bacterial and fungal growth at the recommended concentration of 10 mL/L. Larvae were stored for 24 hours, in a dark cabinet until the morning of gavage (6 days post-irradiation) when larvae were isolated into 1.5-mL microcentrifuge tubes, 50 larvae in 500 *µ*L of tap water per tube, and 14 tubes were made per treatment. These larvae were then used to infect rats. Four extra tubes were made for genetic analysis to verify that *A. cantonensis* was present in the pool of larvae from each treatment. The remaining larvae, roughly 600 larvae per treatment, were transferred into a 384-well plate, along with methanol-frozen dead controls, and monitored for mortality using the propidium iodide assay analysis as described earlier. These larvae were not monitored for motility after the propidium iodide assay as were the in vitro larvae.

### Experimental infection of rats with irradiated *A. cantonensis*.

Fifty-six outbred Wistar IGS laboratory rats (*Rattus norvegicus*) (Charles River Laboratory Inc, Raleigh, NC) were housed at the USDA-Animal Plant Health Inspection Service-National Wildlife Research Center (USDA-APHIS NWRC) Hawaii Field Station, Hilo, HI. Upon arrival, 8-week-old rats were weighed and allowed to acclimate for 7 days. Throughout the study, rats were maintained in individual polycarbonate shoebox cages, with ad libitum access to water in ball-stoppered bottles, and Laboratory Rodent Diet 5001 (LabDiet, St. Louis, MO) as well as occasional apples and carrots. The room was maintained at a mean temperature of 22.1°C (± 0.02 SE) and a 12:12 h light–dark cycle. With an equal sex ratio, rats were evenly divided across four treatment groups (*n* = 14 per treatment), which included an uninfected water control, 0- (unirradiated larvae), 150-, and 400-Gy larvae. Rats were infected at 13 days after arrival with *A. cantonensis* larvae that were 6 days after semi-slug irradiation by gavaging 500 µL of water either with or without larvae to rats according to their designed treatment groups. Individual, sterile gavage tubes (Instech Laboratories, 1-FTP-15-78 and 1-FTP-15-100) were used to deliver the 500 µL of water with or without *A. cantonensis* larvae to the designated rat.

To facilitate gavage, rats were anesthetized using a Tec III 300P vaporizer (Vaporizer Sales & Services, Rockmart GA) to administer a mixture of isoflurane-oxygen gas. To reduce exposure of personnel to isoflurane, all operations were carried out within a DWS36-A ductless fume hood (Air Science DWS Downflow Workstation, Fort Myers, FL). Initial anesthetization of each rat took place with a sealed APHIS-constructed plexiglass induction chamber with an oxygen flow rate of 2 to 3 LPM and isoflurane concentration of 2–4%.

After collection of final weight data, all rats were humanely euthanized in a CO_2_ chamber and necropsied. Seven rats (four females, three males) from each treatment (50%) were euthanized at 3 weeks (23–24 days) PI, and the remaining rats euthanized (three females, four males) at 6 weeks (44–45 days) PI. Dissection tools, trays, and tube racks for necropsy were decontaminated by a 10% bleach (Clorox 8.3% v/v sodium hypochlorite diluted 1:10) for 20 minutes, thoroughly rinsed under running tap water, and both sides of the tools exposed to UVC irradiation for either 20 minutes in a UV Crosslinker (UVP, CX-2000) or 60 minutes in a biosafety cabinet (Thermo Fisher Scientific, 1387).^27^ Decontaminated tools were then packaged and sealed as sets in pouches (Fisher Scientific, 01-812-57), which included two forceps, a scalpel handle and blade, dissection scissors, pins, and a new plastic Petri dish. To avoid cross-contamination within and between rats, two clean sets of tools were used for each rat, one was used for the heart, lung, and feces collection and a second set was used for the brain dissection. Tissues were examined both externally and internally for *A. cantonensis* under microscopy using either a Leica or an Olympus stereoscope (10–50×). Adult *A. cantonensis* found in the heart/lung complex were placed in 200 µL of DNA lysis buffer (0.1M Tris-HCL, 0.1M EDTA, 2% SDS). Roughly equal proportions of the pulmonary artery, right ventricle, and upper right lobe of the lung were placed in a 2.0-mL microtube with six 2.3-mm zirconia/silica beads (BioSpec Products 11079125z), 0.2 g of 0.5 mm zirconia/silica beads (BioSpec Products, 11079105z), and 500 µL of DNA lysis buffer for bead mill homogenization. The frontal lobe of the brain and any whole larvae observed were placed in 2.0-mL microtubes as described above for bead mill homogenization. Feces were collected for only the 6-week euthanized rats and placed into empty 2.0-mL microtubes. All tissues were frozen at –80°C until processing.

Rats were imported under the State of Hawaii Department of Agriculture permit 22-05-H-L7086a, and all procedures in this study were approved by Institutional Animal Care and Use Committees (USDA-APHIS NWRC protocol QA-3346 and University of Hawaii protocol 21-3576) and the University of Hawaii Biosafety Committee (protocol 21-03-505-04), in accordance with the Guidelines of the American Society of Mammologists for the use of mammals in research.^28^

### Genetic analyses.

Brain tissue from the 6-week necropsy was thawed on ice and homogenized using a Bead Ruptor Elite (Omni International, 19-2241E, Kennesaw, GA) for three cycles of 8,000 m/s for 1 minute, ice for 5 minutes, and centrifuged at 6,200 g for 3 minutes. DNA was extracted from the larvae and 100 µL of the homogenized brain samples using the Dneasy Blood and Tissue Kit (Qiagen, 69504, Venlo, The Netherlands) per the respective manufacturer’s protocol with final elution volumes of 100 µL for larvae and 400 µL for brain tissue. DNA was extracted from the feces using the QIAamp Fast DNA Stool Mini Kit (Qiagen, 51604) per the manufacturer’s protocol with 100-µL final elution volume. PCR reactions were run on a QuantStudio 5 Real-Time PCR Instrument (A28134, Applied Biosystems, Waltham, MA) with a species-specific multiplex assay, TaqMan Fast Advanced Master Mix (4444965, Applied Biosystems), and the recommended cycling conditions with the exception of using 50 instead of 40 cycles (L. M. Kaluna and S. I. Jarvi, unpublished data). Samples were analyzed using a 0.05 manual threshold and designated as positive based on the cycle threshold (C_T_ value ≤40), negative (C_T_ value 0), or equivocal (where replication of a positive PCR result was not demonstrated).

## STATISTICAL ANALYSES

Statistical analyses were performed to investigate significant differences between irradiation treatments for 1) the number of live larvae after the 5 minutes of freshwater incubation in the in vitro experiments, 2) the number of live larvae following pepsin-HCl treatment in the in vitro experiments, 3) the number of larvae found in the brain of rats 3-week PI, 4) the number of adult worms found in the heart-lung complex of rats at 6 weeks PI. For statistical analysis of rat necropsy data, only data from the infected treatment groups were used (all water control rats were excluded from the analysis). Because most of the data was not normally distributed or did not have equal variances, nonparametric Kruskal-Wallis tests were used for analysis. A one-way analysis of variance (ANOVA) was used for comparisons in the number of live larvae per treatment following a 5-minute incubation period in fresh water, and a two-way ANOVA was used to evaluate percent body weight gain among treatments in male and female rats at 3 and 6 weeks PI. Two-sample *t* tests were used to compare the number of *A. cantonensis* worms in the brain at 3 weeks and the heart/lung complex at 6 weeks after normality testing. Analyses were performed in Minitab v. 21 and a significance level of 0.05 was used.

## RESULTS

### In vitro irradiation of L3 *A. cantonensis*.

**Figure 1.**
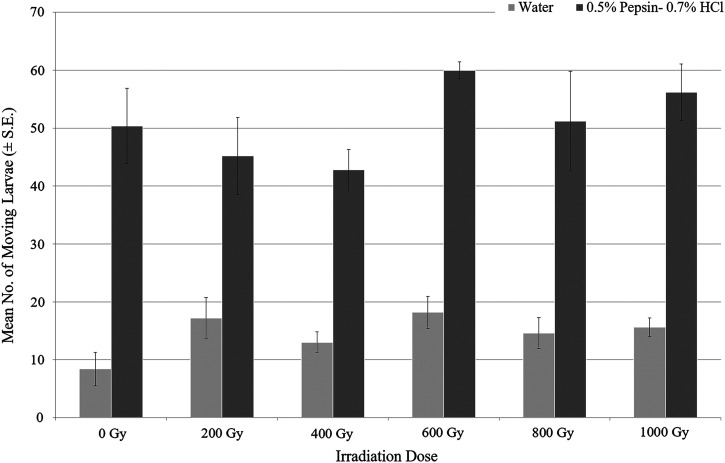
After in vitro x-ray irradiation exposure, the number of actively swimming *Angiostrongylus cantonensis* larvae (mean ± SE) at 27 days post-irradiation. No significant differences were found across treatments after five minutes of incubation in fresh tap water (*P* = 0.155) or 0.5% pepsin-0.7% HCl solution (*P* = 0.382).

To determine the effects of x-ray exposure on *A. cantonensis* larvae, 200 larvae were directly exposed to irradiation at doses of 0, 200, 400, 600, 800, and 1,000 Gy. Mortality of *A. cantonensis* larvae remained very low (<3.5%) for all irradiation doses up to 23 days postirradiation, with no observed dose-response. After 27 days postirradiation, a mean of eight to 18 larvae (4–9%) per treatment replicate were actively swimming in fresh tap water, which increased to 42 to 60 larvae (21–30%) per treatment replicate after exposure to the pepsin-HCl solution (Figure 1). Although exposure to pepsin-HCl clearly resulted in greater activation of the larvae, no significant differences were found across treatments for either the number of moving larvae after the 5-minute fresh tap water incubation (*P* = 0.155) or the pepsin-HCl solution (*P* = 0.382). The physiological state of the unstained, nonmobile larvae is unknown. Genetic analysis confirmed the presence of *A. cantonensis* DNA in sample pools used for testing.

### In vivo irradiation of *P. martensi* (semi-slugs) for *A. cantonensis* isolation.

Similar to the in vitro irradiation propidium iodide assay results, there was almost no mortality observed (≤1.0%) up to 26 days postirradiation of *A. cantonensis* larvae isolated from semi-slugs exposed to x-ray irradiation for all irradiation doses (0, 150, and 400 Gy). All samples of nematodes saved for genetic analysis of each treatment were positive for *A. cantonensis* DNA.

### Experimental infection of rats with irradiated *A. cantonensis*.

**Figure 2.**
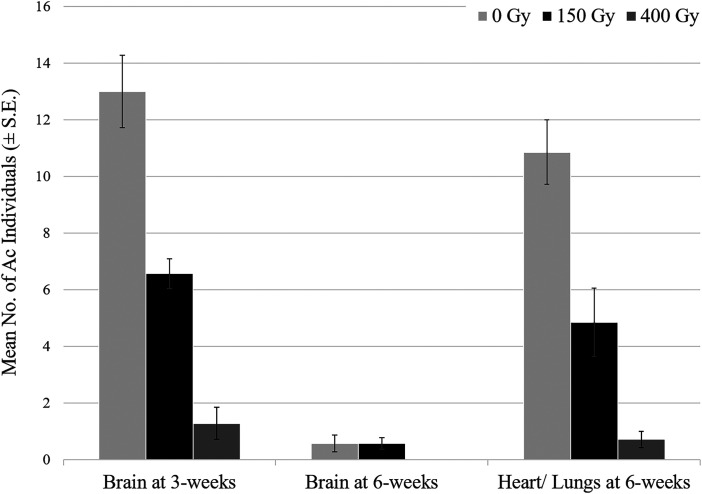
The number of *Angiostrongylus cantonensis* larvae or adults (mean ± SE) observed upon necropsy at either 3 or 6 weeks postinfection with larvae isolated from infected *Parmarion martensi* exposed to 0-, 150-, or 400-Gy irradiation. Significant differences were observed between treatments in the number larvae detected in the brain at 3 weeks (*P* = 0.000), and adults detected in the heart/lung complex at 6 weeks (*P* = 0.000). No significant differences were found when comparing the number larvae in the brain with the number of adults in the heart/lung complex within the 0 Gy (*P* = 0.236), 150 Gy (*P* = 0.228), or 400 Gy (*P* = 0.393) treatments.

An equal number (50%) of rats in each treatment was humanely euthanized at 3 PI (*n* = 7), and the remaining seven rats were euthanized at 6 weeks PI. Larvae were observed in the brain at 3 weeks in all rats infected with 0-Gy larvae (7/7) and 150-Gy larvae (7/7) and most rats (4/7) infected with 400 Gy larvae. Whereas the number of rats with larvae observed in the brain at 6 weeks PI was three of seven in the 0-Gy treatment, four of seven in the 150-Gy treatment, and zero of seven in the 400-Gy treatment. The mean number of *A. cantonensis* larvae observed in the brain at 3 and 6 weeks PI, and the mean number of adult worms found in the heart-lung complex are shown in Figure 2. Significant differences were observed between infected treatment groups in both the number of larvae observed in the brain upon necropsy at 3 weeks (*P* = 0.000) and the heart-lung complex at 6 weeks (*P* = 0.000), with a clear decline corresponding with increasing irradiation dose. As expected, no larvae were detected in the heart–lung complex at 3 weeks (data not shown), and few to no larvae were detected in the brain at 6 weeks. No significant differences were found when comparing the number of *A. cantonensis* larvae found in the brain at 3 weeks verses adults in the heart–lung complex at 6 weeks in the 0 Gy (*P* = 0.236), 150 Gy (*P* = 0.228), or 400 Gy (*P* = 0.393) treatments. The mean number of larvae found in the brain tissue at 3 weeks was 13 (± 3 SD), 7 (± 1 SD), and 1 (± 1 SD) for the 0, 150 y, and 400 Gy treatments, respectively. Similarly, the mean number of adults found in the heart–lung complex at 6 weeks was 11 (± 3 SD), 5 (± 3 SD), and 1 (± 1 SD), in each respective treatment. Rat body weights were recorded at the start of the study, and at the 3- and 6-week necropsies. Males had significantly higher body weight at the start of the study than females (*P* <0.0001) (Supplemental Table 1). Mean percent body weight gain at 3 and 6 weeks for females and males was 39.6 (± 8.0 SD) and 57.6 (± 5.5 SD), and 50.0 (± 8.9 SD) and 86.9 (± 11.6 SD), respectively. The effect of treatment on percent weight gain was not significant at 3 weeks (*P* = 0.31) or 6 weeks (*P* = 0.75).

Details of infection within individual rats necropsied at 6 weeks are presented in Table 1, including the number of adult worms and presence of eggs or L1 found in the heart–lung complex, as well as the presence of L1 stage found in the feces. All L1 larvae present in the lungs and feces at 6 weeks PI were actively moving. No eggs or L1s were visually detected in the feces of the 150- and 400-Gy treatment groups. Also, in Table 1 the results of PCR detection of *A. cantonensis* DNA in brain tissue and feces at the 6-week necropsy are given. No amplification was seen in brain samples from control rats that received just water, but PCR amplification was observed among all rats (seven of seven) infected with larvae exposed to 0 Gy irradiation, five of seven rats infected with larvae exposed to 150-Gy irradiation, and six of seven rats infected with larvae exposed to 400-Gy irradiation. PCR amplification from fecal samples was not detected in the water control rats, nor rats subject to exposure to 150- and 400-Gy levels of irradiation, but amplification was detected in rats gavaged with larvae exposed to 0-Gy irradiation, indicating lack of reproduction among irradiated *A. cantonensis* larvae regardless of dose.

**Table 1:** Rats necropsied at 6 weeks postinfection; identification number (ID), sex, treatment, the observable number of adult worms found in the heart/lung complex, larvae present in lungs and feces, and real-time PCR results of brain tissue and fecal samples

			Necropsy Observations			Real-Time PCR Results							
						Brain Tissue				Feces			
Rat ID	Sex	Treatment	No. Adult Worms in Heart/Lungs	Eggs or L1s Present in Heart/Lungs	L1s Present in Feces	Mean C_T_	C_T_ SD	No. Pos. Reps	Result	Mean C_T_	C_T_ SD	No. Pos. Reps	Result
Rat_025	F	Water	0	No	No	0.00	–	0	N	0.00	–	0	N
Rat_026	M	Water	0	No	No	0.00	–	0	N	0.00	–	0	N
Rat_027	F	Water	0	No	No	0.00	–	0	N	0.00	–	0	N
Rat_028	M	Water	0	No	No	0.00	–	0	N	0.00	–	0	N
Rat_098	M	Water	0	No	No	0.00	–	0	N	0.00	–	0	N
Rat_101	M	Water	0	No	No	0.00	–	0	N	0.00	–	0	N
Rat_108	F	Water	0	No	No	0.00	–	0	N	0.00	–	0	N
Rat_017	F	0 Gy	7	Yes	No	33.22	0.96	3	P	18.77	0.06	3	P
Rat_018	M	0 Gy	10	Yes	No	29.30	0.20	3	P	19.36	0.07	3	P
Rat_019	F	0 Gy	13	Yes	Yes	26.06	0.10	3	P	16.13	0.12	3	P
Rat_020	M	0 Gy	12	Yes	No	18.49	0.04	3	P	20.87	0.04	3	P
Rat_046	F	0 Gy	9	Yes	No	33.38	0.49	3	P	30.17	0.11	3	P
Rat_051	M	0 Gy	16	Yes	Yes	26.57	0.07	3	P	19.85	0.02	3	P
Rat_053	M	0 Gy	9	Yes	Yes	18.96	0.13	3	P	25.82	0.05	3	P
Rat_001	F	150 Gy	1	No	No	0.00	–	0	N	0.00	–	0	N
Rat_002	M	150 Gy	8	No	No	29.70	0.46	3	P	0.00	–	0	N
Rat_003	F	150 Gy	2	No	No	17.53	0.04	3	P	0.00	–	0	N
Rat_004	M	150 Gy	10	No	No	17.81	0.05	3	P	0.00	–	0	N
Rat_036	M	150 Gy	5	No	No	37.08	1.81	2	P	0.00	–	0	N
Rat_037	M	150 Gy	4	No	No	18.27	0.02	3	P	0.00	–	0	N
Rat_038	F	150 Gy	4	No	No	0.00	–	0	N	0.00	–	0	N
Rat_009	F	400 Gy	1	No	No	39.54	1.72	2	P	0.00	–	0	N
Rat_010	M	400 Gy	2	No	No	37.90	–	1	E	0.00	–	0	N
Rat_011	F	400 Gy	1	No	No	38.41	2.01	3	P	0.00	–	0	N
Rat_012	M	400 Gy	0	No	No	21.44	0.07	3	P	0.00	–	0	N
Rat_041	M	400 Gy	0	No	No	32.46	0.07	3	P	0.00	–	0	N
Rat_045	F	400 Gy	1	No	No	31.28	0.03	3	P	0.00	–	0	N
Rat_047	M	400 Gy	0	No	No	39.26	3.78	2	P	0.00	–	0	N

PCR = polymerase chain reaction. Mean cycle threshold (C_T_) and cycle threshold standard deviation (C_T_ SD) are reported. All real-time PCR samples were tested in triplicate, with results of negative (N), positive (P), or equivocal (E).

**Figure 3.**
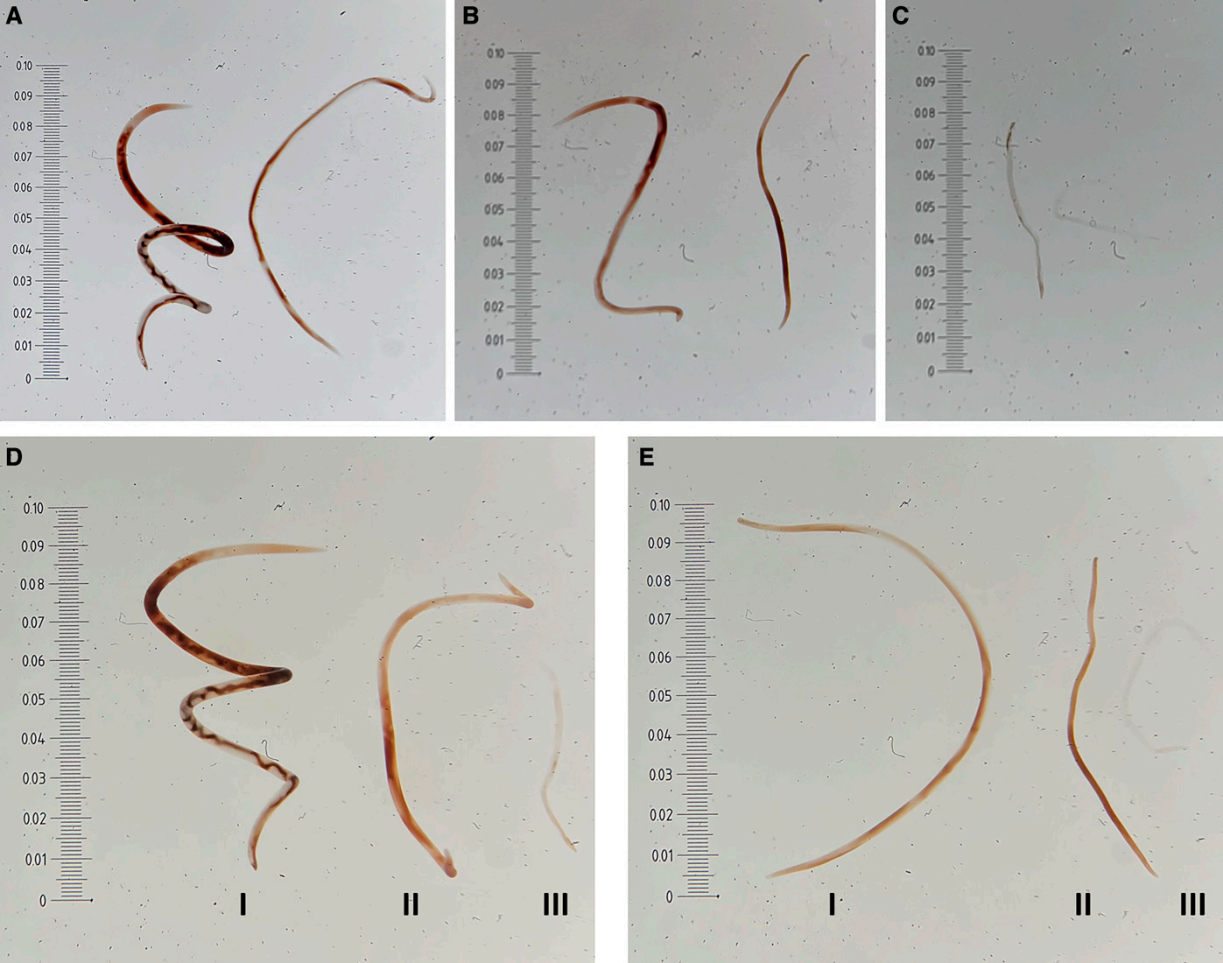
Morphological abnormalities of adult *Angiostrongylus cantonensis* in response to x-ray irradiation as third-stage larvae. Sex determination was surmised for irradiated worms based on the relative size of worms within a treatment, where nonirradiated female worms are significantly larger than male worms. (**A**) Adult 0-Gy treated female (left) and male (right) worms with exoskeletons that are rigid and well-defined, dark brown intestine, female intertwined uterus and intestine. (**B**) Adult 150-Gy treated female (left) and male (right) worms with slight discoloration, growth retardation, flaccid exoskeleton, and less defined intertwined uterus and intestine in females. (**C**) Adult 400-Gy treated female (left) and male (right) worms, with severe discoloration, growth retardation, flaccid exoskeleton, and no intertwined uterus and intestine in females. (**D**) Size comparison of female adult worms between (I) 0, (II) 150, and (III) 400 Gy x-ray irradiation. (**E**) Size comparison of male adult worms between (I) 0, (II) 150, and (III) 400 Gy x-ray irradiation. Note: Images of adult worms were captured using a Leica S9 dissection microscope at 1.6× magnification with a Leica Reticule 100/0.001 attached to the eyepiece.

Additional observations were made regarding morphological abnormalities, where we observed but did not quantify that all adult worms recovered from both irradiated treatments exhibited retarded growth, discoloration, flaccidity, and sluggish motility in a dose–response manner. Sex determination in irradiated worms was challenging due to the absence of distinct intertwined uterus and intestine (Figure 3). The sex of adult worms was inferred based on the relative size of worms within a treatment, where female adult worms are substantially larger compared with male adult worms.^29^

## DISCUSSION

Phytosanitary treatments such as irradiation are applied to fresh agricultural commodities to prevent the introduction and spread of quarantine or regulated pests and are often the simplest measure to overcome regulatory trade barriers and gain market access.^30^ Global trade volume of irradiated produce has increased nearly 10-fold from 2007 to 2019 to ∼47,000 tonnes.^31^^,^^32^ The volume of tropical fruits exported to the United States from foreign countries has grown steadily, with the main fruits including mangos (India, Pakistan, Mexico, and Australia); guava (Mexico), persimmons (South Africa); mangosteen (Thailand); and dragon fruit, longan, and rambutan (Vietnam). Domestically, Hawaii is exporting 5–6 tonnes of tropical fruits and vegetables to the United States mainland annually, with sweet potato being the main export. Australia recently began exporting a variety of irradiated fruits to New Zealand and several Southeast Asian countries and is currently the largest exporter of irradiated fresh produce. Irradiation is approved to treat fresh fruit for export in several countries where *A. cantonensis* has been confirmed including India, Thailand, China, Laos, and Dominican Republic.^6^^,^^15^

Results from this study show that although x-ray irradiation does not result in the direct mortality of *A. cantonensis* larvae, it does affect its ability to infect a rat (its definitive host) as well as its reproduction within the host if infection is successful. This is likely due to the energy imparted to the target by the absorbed radiation which causes chemical bond breakage including in the DNA molecule. DNA changes result in disruption of normal cell physiology that leads to an inability to reproduce or the death of the target.^33^ In this study, the doses applied did not result in immediate death nor completely inhibit growth of the parasite. It has been shown that different stages of the insect life cycle have different tolerances (resistance) to radiation damage and different insects also display different tolerances.^15^ Phytosanitary irradiation protocols are designed to prevent reproduction through the prevention of adult emergence or through adult or F1 generation sterility, which is what we report in this study. Higher irradiation doses that guarantee near-immediate mortality (in insects) and life stages could be applied but tend to have adverse effects on the sensory qualities of fresh produce, and in some cases might require doses that exceed the 1,000-Gy maximum allowable dose.^15^

Our necropsy and PCR results of brain tissue are consistent with results from Pai et al.^22^ and Ooi et al.,^23^ which show nearly all infected rats had some level of infection with a significant, negative dose response observed in the number of worms that migrated to the brain compared with non-irradiated controls (0 Gy). Despite these findings, it remained unclear whether irradiation causes direct, but delayed mortality of *A. cantonensis*. As was reported by Pai et al.,^22^ we also observed motility in larvae following irradiation, thus irradiation of doses ≤1,000 Gy is not immediately fatal to larvae. In our in vitro and in vivo irradiation experiments, results showed very low mortality and no dose response across all larval irradiation treatments 27 days postirradiation. Therefore, irradiation of larvae, isolated or within a snail, does not directly cause mortality of the parasite over the timespan that it would be expected to take for larvae to invade the central nervous system of a host (∼4 hours PI to invade and ∼2 weeks for young adults to appear on the brain).^29^

Although irradiation is not fatal to *A. cantonensis* directly, decreased infection levels in brain tissue of definitive and nonpermissive hosts across multiple studies indicate irradiation does have a deleterious effect on a larva’s migration to the brain. Irradiated larvae are unable to migrate to the central nervous system, are more susceptible to the host immune response during migration, or both. In fact, even low doses of irradiation (100 Gy) have been shown to affect larvae’s motility,^34^ yet it remains unclear how exactly this could affect the migration from the gastrointestinal tract to the brain. Regardless of the mechanism, greatly reduced brain infection from irradiated larvae in both definitive and nonpermissive hosts is promising. A positive correlation between parasite burden and the severity of symptoms is clear, as has been shown in rabbit models^35^^,^^36^ (a nonpermissive host), in the definitive rat host,^37^ and is additionally supported by human clinical evidence.^38^ Although theoretically the consumption of infected, irradiated, exported produce may result in neuroangiostrongyliasis, the severity of the infection should be greatly reduced due to fewer larvae penetrating the brain.

Similar to brain infection results, a negative dose response was found in the number of adult worms in the heart–lung complex at 6 weeks in the irradiated treatments. Within each treatment, there were no significant differences in the mean number of adults found in the heart–lung complex at 6 weeks compared with the mean number of larvae found in the brain at 3 weeks (Figure 2). This suggests most larvae that successfully migrated to the brain were able to develop to the adult stage and migrate to the heart and lungs of the rat. Qualitative morphological observations were consistent with Ishii et al.^34^ and Ooi et al.^23^ in finding irradiation caused retarded growth and a lack of the “barber’s pole” pattern of intertwined uteri and intestine in females, however, we also observed discoloration and flaccid exoskeletons (Figure 3). Necropsy and PCR results also show that irradiated adult worms, detected in the pulmonary arteries of rats, were not able to produce eggs or L1 larvae. Parasite DNA was only detected in the feces of rats gavaged with larvae exposed to 0-Gy irradiation. Therefore, irradiation doses ≥150 Gy effectively sterilized *A. cantonensis*, thus rendering it unable to complete its life cycle in rats. These findings are consistent with previous research, where irradiation doses of 100–250 Gy prevented reproduction in rats.^20^^,^^22^^,^^34^ Thus, the USDA generic dose of 150 Gy should preclude irradiated *A. cantonensis* from establishing viable populations in export destinations thereby reducing the potential for global spread and provide quarantine security.

## CONCLUSION

Results from our study found a significant negative dose effect in the infectivity of *A. cantonensis* to the phytosanitary irradiation doses of 150 and 400 Gy, as well as both doses precluding reproduction in adult worms. With irradiated *A. cantonensis* larvae being unable to produce eggs within their definitive host, feces from infected rats would be sterile and unable to infect snails, and the *A. cantonensis* life cycle is interrupted. Although it may still be possible that irradiated larvae could cause neurological disease in humans or other accidental hosts, *A. cantonensis* will be unable to establish viable populations from *P. martensi* (and likely other snail species) in export destinations. The long-term effects of a reduction in transmission of the parasite to intermediate hosts on the overall parasite population remain to be determined.

## Supplemental Materials

10.4269/ajtmh.23-0570 Supplemental Materials
