# Supplementary material for: Efficacy of X-Ray Phytosanitary Irradiation on the Infectivity and Reproduction of Angiostrongylus cantonensis in Experimentally Infected Rats
Source: Am J Trop Med Hyg. 2024 Feb 20;110(4):691–9. doi: 10.4269/ajtmh.23-0570 (PMC10993834; doi:10.4269/ajtmh.23-0570)
Supplement: Supplemental Materials [file tpmd230570.SD1.pdf]

Supplemental Table 1

Mean initial and final weights (g) of rats per treatment at the three- and six-week necropsy. The effect of treatment on weight gain was not significant at 3 weeks ($P = 0.31$) or 6 weeks ($P = 0.75$)

Treatment	Age at necropsy	*n*	Sex	Mean weight (g)		Weight change (g)
				Initial	Final	
Water	3 weeks	4	F	168.0	243.5	75.5
		3	M	238.3	378.7	140.3
0 Gy L3	3 weeks	4	F	173.5	235.5	62.0
		3	M	243.7	380.0	136.3
150 Gy L3	3 weeks	4	F	174.5	244.0	69.5
		3	M	252.3	402.3	150.0
400 Gy L3	3 weeks	4	F	170.3	233.8	63.5
		3	M	242.3	377.7	135.3
Water	6 weeks	3	F	153.3	240.0	86.7
		4	M	247.0	457.5	210.5
0 Gy L3	6 weeks	3	F	167.0	235.3	68.3
		4	M	234.0	448.3	214.3
150 Gy L3	6 weeks	3	F	164.3	247.0	82.7
		4	M	249.5	469.8	220.3
400 Gy L3	6 weeks	3	F	166.3	251.3	85.0
		4	M	266.0	479.8	213.8
